# Ethyl Carbamate Analysis in Fermented Products: A Comparison of Measurements of Mass Spectrometry, Thermal Energy Analyser, and Hall Electrolytic Conductivity Detector

**DOI:** 10.6028/jres.093.033

**Published:** 1988-06-01

**Authors:** M. J. Dennis, N. Howarth, R. C. Massey, D. J. McWeeny, I. Parker, M. Scotter, J. R. Startin

**Affiliations:** Food Science Laboratory, Ministry of Agriculture, Fisheries and Food, Norwich and London, U.K.

## 1. Introduction

Ethyl carbamate occurs in some alcoholic beverages in concentrations ranging from < 10 μg/L to >1000 μg/L. Typically, the concentration is low in wine and high in some spirits; the concentration is particularly high in some plum brandies but is low in gin and vodka. Literature methods [1,2,3] rely on sample clean-up followed by packed- column GC and detection by FID, alkali flame ionisation, electron capture, or Coulson electrolytic conductivity detector and allow measurements down to 10 μg/L. The clean-up procedure required for levels below 100 μg/L is extensive and MS confirmation is still required.

In the course of a study of ethyl carbamate levels in alcoholic beverages on sale in U.K., different methods of separation and measurement were assessed. Using extracts prepared by the clean-up procedure described below the three measurements were carried out within MAFF Food Science Laboratory over a period of some weeks by separate groups using respectively GC-Hall Electrolytic Detector, GC-Thermal Energy Analyser and GC- Mass Spectrometry. Results are shown in table 1.

## 2. Extraction and Clean-Up Procedure

Samples were diluted to <5% alcohol and 50 mL passed down a Chemtube (CT 2050, Analytichem) or Extrelut (Merck, 42g). Ethyl carbamate was eluted with 3×50 mL dichloromethane, the extract was dried on a sodium sulphate column and concentrated to 4 mL prior to passage down a Florisil Sep-Pak (Waters) pre-rinsed with dichloromethane. The Sep-Pak was rinsed with dichloromethane and ethyl carbamate was eluted with 7% (V/V) methanol in dichloromethane (5 mL) and concentrated to about 0.7 mL in a micro Kuderna-Danish evaporator. Final volume was measured by syringe. Average recoveries were 84% (minimum 75%).

## 3. GC Conditions

Slightly different gas chromatographic conditions were employed for each detector to gain optimum performance from each instrument. The following, used for the thermal energy analyser (TEA) detector, was typical. CP Wax 52 CB column (Chrompack), 25 m × 0.31 mm I.D., 0.21 μm Film thickness, temperature programme 65 °C (for 1 min) then 10 °C/min to 100 °C and 20 °C/min to 200 °C (for 10 min).

On-column injection (2 μL) with secondary cooling was employed with helium carrier gas (0.6 bar).

## 4. Detector Operating Conditions

Hall 700A Electrolytic Conductivity Detector (Tracor) was used in nitrogen-mode without scrubbing; furnace 840 °C; isopropanol:water (1:1) electrolyte at 0.4 mL/min with manual venting; hydrogen flow 40 mL/min.

Thermal Energy Analyser Model 610 (Thermedics) was used in the nitrogen mode; interface temperature 200 °C; furnace 800 °C; reaction chamber pressure 2 mm Hg; no make-up gas.

VG 7070H mass spectrometer was used in E.I. mode (70 eV, 200 μA trap current, 200 °C) with interface heater at 200 °C. Selected ions were monitored (*m/z* 61, 62, 74).

## 5. Discussion

The Chem-Tube/Extrelut clean-up saved much time in clearing dichloromethane:water emulsions. The Florisil Sep-Pak allowed further clean-up in about 1 minute and although not strictly required for TEA purposes was necessary if a full mass spectrum is required for confirmatory purposes and greatly extends capillary column life.

**Figure 1:**
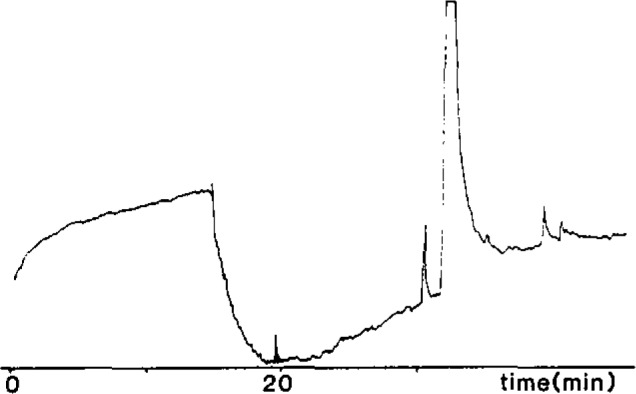
Capillary GC analyses of ethyl carbamate in whiskey using Hall 700A electrolytic conductivity detector. Shadowed area, 32 μg/L.

Hall Electrolytic Conductivity gave a high background signal due to HCI from dichloromethane; this could be avoided by changing the solvent to ethyl acetate or (more conveniently) by manual venting of the detector until just before ethyl carbamate elutes. The scrubber system was not used as it adversely affected peak width without significantly reducing background noise. Instrument behaviour was variable and optimum performance demanded considerable operator skill. Figure 1 shows a typical chromatogram.

The mass spectrum of ethyl carbamate is shown in figure 2(a). Monitoring *m/z* 62 provided high selectivity and a 1 μg/L detection limit. The relative heights of *m/z* 62, 61, and 74 were used to confirm identity/purity. A typical chromatogram, monitoring *m/z* 62 is shown in figure 2(b).

**Figure 3:**
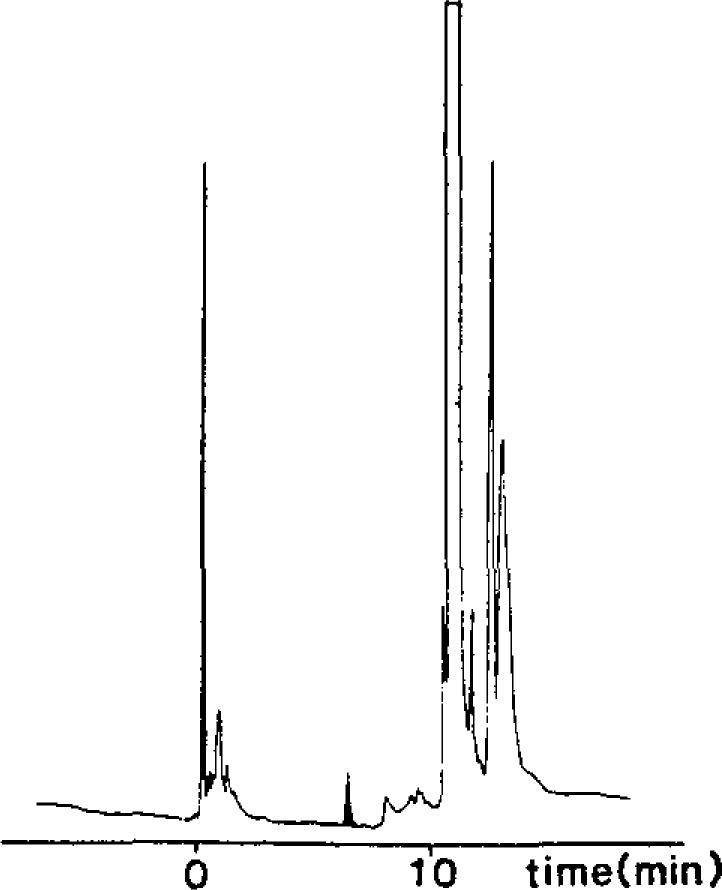
Capillary GC analysis of ethyl carbamate in whiskey using thermal energy analyser (nitrogen mode). Shadowed area 43 μg/L.

The Thermal Energy Analyser, like the Hall, showed relatively few peaks but gave very stable performance (fig. 3). Blanks were consistently below the detection level (1 μg/L); duplicates were within 5% of the mean. Duplicate analyses on three separate days showed a coefficient of variation of 6.34% for the six analyses of a bourbon whiskey containing 300 μg/L ethyl carbamate.

## 6. Conclusions

A rapid clean-up procedure followed by GC- TEA separation and measurement suitable for routine analysis of ethyl carbamate down to 1 μg/L in alcoholic beverages has been developed. Results correlate well with data from alternative detector systems. Fuller details are reported elsewhere [4].

## Figures and Tables

**Figure 2(a):**
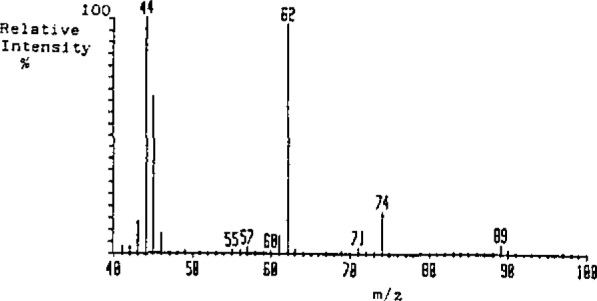
Electron impact spectrum of ethyl carbamate.

**Figure 2(b):**
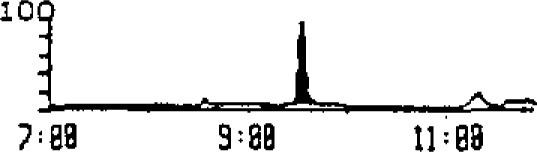
Capillary GC-MS analysis (*m/z* 62) of ethyl carbamate.

**Table 1:** Comparison of results for ethyl carbamate (μg/L) obtained from different detectors

Sample	Mass spectrometer (*m/z* 62)	TEA detector	Hall detector
Bourbon Whiskey a^a^	216^a^	204	176
b	212^b^	208	184
Scotch Whiskey a^a^	75	80	72
b	77	99	84
Red Wine a	22	16	13
b	Not analysed	13	10

a Each sample was extracted and concentrated in duplicate (a and b). Each concentrate was then analysed by the different detectors.

b Confirmed from *m/z* 61, 74.
